# The Significance of Proprioceptive Training in the Post-Operative Rehabilitation of Patients Undergoing Achilles Tendon Reconstruction

**DOI:** 10.7759/cureus.54431

**Published:** 2024-02-18

**Authors:** Snehal Samal, Subrat Samal, Vaishnavi M Thakre

**Affiliations:** 1 Neuro Physiotherapy, Ravi Nair Physiotherapy College, Datta Meghe Institute of Higher Education & Research, Wardha, IND; 2 Musculoskeletal Physiotherapy, Ravi Nair Physiotherapy College, Datta Meghe Institute of Higher Education & Research, Wardha, IND

**Keywords:** pnf, proprioception, ankle joint, rehabilitation, achilles tendon injury

## Abstract

In the human anatomy, the Achilles tendon (AT) is the strongest and largest tendon. Also, it ruptures the most often. Because it impairs the patient’s ability to function adequately, a ruptured AT injury is a serious clinical issue. Reconstruction of the tendon through surgical intervention is the preferred approach to treatment in the case of tendon rupture. Establishing an effective post-operative rehabilitation regimen that mostly consists of functional physiotherapy measures is crucial in the management of AT rupture. In this report, we have presented the case of an AT reconstruction patient who complained of pain in the ankle region, reduced strength and range of the ankle joint, and loss of proprioception. The tailor-made physiotherapy protocol was incorporated, which included strengthening exercises, proprioceptive retraining, cryotherapy, and ambulatory training, which were found to be effective in facilitating early functional recovery.

## Introduction

The calcaneal tendon, commonly known as the Achilles tendon (AT), is a strong band of fibrous tissue that attaches the calf muscles to the calcaneus and is essential for postural control, walking, and running [1]. The gastrocnemius and soleus muscles blend their tendinous contributions to make the AT, which is the strongest and largest tendon in the human body [2]. Regardless of this, in the lower extremities, the AT sustains injuries the most frequently [3]. AT rupture tends to occur in up to 37 out of every 100,000 people per year [4]. There is a bimodal distribution based on age, with women aged 60-80 forming the second peak and men aged 30-40 making up the first. Sports participation, such as racquet and football sports, frequently corresponds to the first peak in incidence, while everyday activities like climbing stairs are often associated with the second peak [5]. Partial or total ruptures may take place. A total rupture results in severe pain and an abrupt loss of strength and movement in the affected limb, but a partial rupture may be asymptomatic or cause only minor symptoms. In addition, ruptures can be classified as acute traumatic ruptures or chronic or neglected ruptures, which occur when an injury goes untreated for longer than four weeks [6].

There is a lack of clarity regarding the etiology of AT ruptures. Numerous factors, including sex, age, changes in training patterns, poor technique, prior injuries, degeneration, poor tendon vascularity, a suboptimally conditioned musculotendinous unit, dysfunction of the gastrocnemius-soleus, and footwear, have been associated with AT ruptures. The “degenerative theory” and the “mechanical theory” are the two fundamental theories that are recognized. The degenerative theory states that without applying excessive loads, a rupture results from chronic deterioration of the tendon [7]. Additional tests to confirm the diagnosis after the examination and palpation are advised. Even though the Simmonds test has a good track record overall, misinterpretation might occasionally occur. The O’Brien and Copeland tests may be used in these circumstances. AT ruptures can be accurately diagnosed by ankle lateral radiography. Ultrasonography is still the primary imaging modality for the AT, even though it is operator dependent [8].

Both nonsurgical and surgical methods can be used to treat acute AT ruptures. For a minimum of four weeks following surgery, conservative treatment comprises immobilization and non-weight-bearing. The correct surgical technique and post-operative care are the two main components of the surgical management of a torn AT [9,10]. The ideal post-operative rehabilitation protocol following surgical repair of an AT rupture is transforming the traditional approach, consisting of six weeks of rigid immobilization in a non-weight-bearing cast below the knee, followed by strengthening and ankle range of motion (ROM) exercises [11]. Proprioceptors are damaged during surgery, and lower limb tendon proprioception impairments are seen. Additionally, prolonged cast immobilization following surgery results in a loss of ankle proprioceptive function. Proprioception allows for the programming of neuromuscular motor control, which is required for precise movement performance and the induction of muscle reflexes that maintain the joint’s dynamic stability [12]. On the other hand, lower extremity immobilization results from both conservative treatment and surgical tendon restoration, both of which diminish physical activity levels. After surgery, the active ROM of the ankle joint is reduced due to six to eight weeks of cast immobilization. Plantar flexion deficits range from 1% to 16%, and in the early post-operative phase, the calf muscle cross-sectional area might decrease by up to 23%. When a muscle is not stretched throughout the immobilization phase, muscular atrophy increases even more; however, this trend is delayed when the muscle is stretched [13].

Choosing an effective post-operative rehabilitation plan that primarily consists of functional physiotherapy components is crucial to treating AT rupture. As such, physiotherapists have limited access to high-quality, evidence-based guidelines to help them opt for the best proprioceptive treatment approaches [14]. This paper aims to evaluate the effects of proprioceptive exercise rehabilitation along with conventional physiotherapy protocols in patients undergoing AT reconstruction to improve ankle joint proprioception, muscle strength, and ROM and reduce pain for early functional recovery.

## Case presentation

Patient information

We outline the case of a 45-year-old female who was brought to our tertiary care hospital to seek management for her condition. The patient was apparently alright five months ago when she sustained an injury to her right ankle region as her foot got stuck in an open wedge, and while trying to free herself, she turned her leg, resulting in a soft tissue injury over the heel region. The patient developed sudden onset pain and swelling in her right ankle and was immediately taken to a local hospital, where she was given medications. The patient took oral medications, but pain relief was not present. Due to persistence, difficulty in walking, and difficulty getting up from a squatting position, the patient came to our tertiary care hospital, where she had undergone certain investigations, like an X-ray and MRI, and was diagnosed with an acute AT rupture on the right side. She underwent an AT reconstruction. After surgical repair, the patient developed complaints of pain in the ankle region, reduced strength and range of the ankle joint, and loss of proprioception, for which a tailor-made physiotherapy regimen was started.

Diagnostic assessment

**Figure 1:**
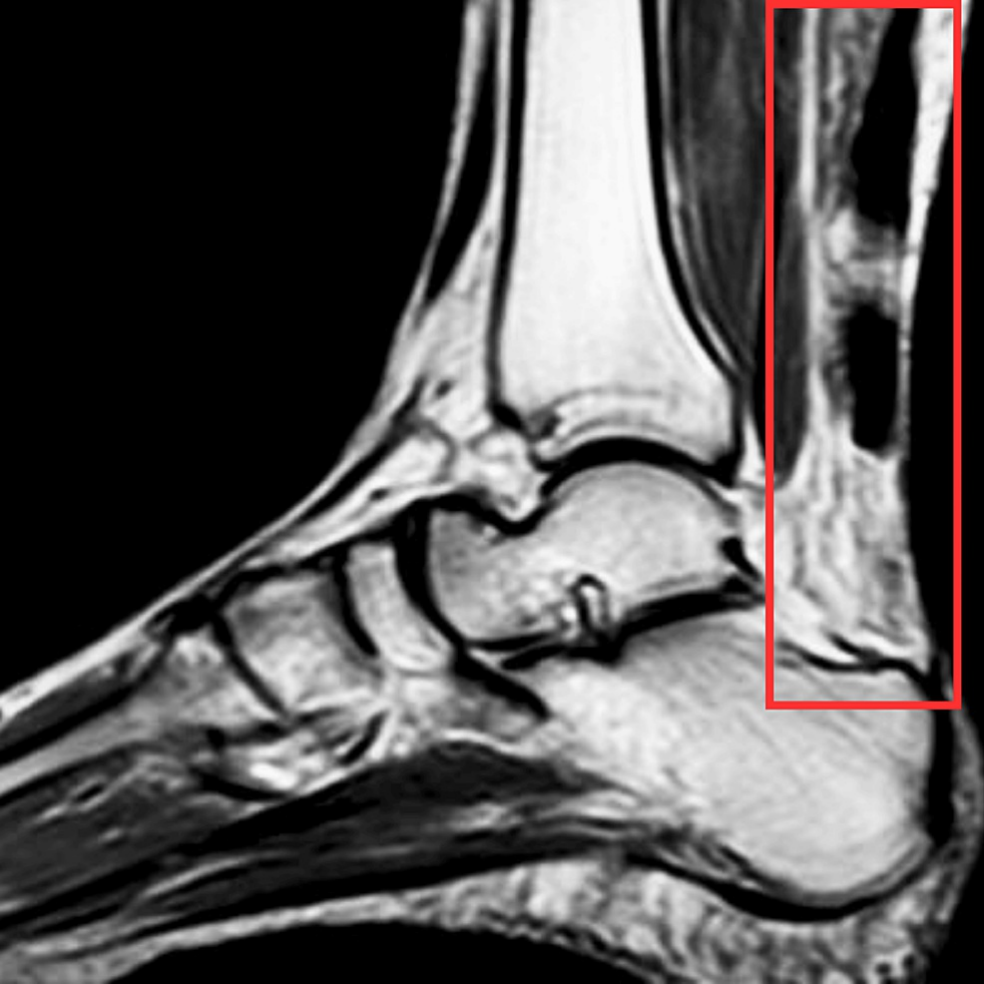
MRI of the ankle joint The rectangle shows a ruptured AT. AT, Achilles tendon

An X-ray examination was done to rule out fracture, and no significant findings were seen. The MRI of the ankle was carried out as a diagnostic tool, which revealed a ruptured AT, as shown in Figure 1.

Physiotherapy assessment

Before the commencement of the examination, informed consent was obtained from the patient. She was cooperative, conscious, and well oriented toward people, places, and time. On examination, the patient was afebrile and hemodynamically stable. The patient was seen in a supine lying posture. She was mesomorphic in build, with a BMI of 23 kg/m^2^. On observation, visible swelling was present near the ankle joint; a vertical scar mark was present on the posterior side of the ankle joint. On palpation, the length of the scar was 8 cm, and according to the tenderness grading system, there was Grade 2 tenderness. The patient complained of pain that was intermittent and dull in nature, and she rated it as 6/10 on activity and 4/10 on rest according to the Numerical Pain Rating Scale. The muscle ROM and strength were significantly reduced at the ankle joint of the right lower limb. The patient was unable to walk independently.

Physiotherapy intervention

The physiotherapist devised particular exercises based on the orthopedician’s loading recommendations and the clinical condition of the patient. The treatment duration was eight weeks. Table 1 depicts the physiotherapy protocol.

**Table 1:** Physiotherapy intervention AAROM, active assisted range of motion; AROM, active range of motion; PNF, proprioceptive neuromuscular facilitation; PROM, passive range of motion

Week	Goals	Intervention	Repetitions
Weeks 1-2 (during plaster cast)	To prevent deep vein thrombosis	Toe curls (affected side) and ankle pumps (unaffected side)	Twenty repetitions × one set (two times per day)
	To maintain the strength of the quadriceps, hamstrings, and glutei muscles	Isometrics: static quadriceps, static glutes, and static hamstrings	Twenty repetitions × one set (two times per day)
	To maintain the strength and ROM of the unaffected limb	AROM exercises for unaffected limb	Ten repetitions × one set (10-second hold)
Weeks 3-8 (after removal of the plaster cast)	To reduce pain	Cryotherapy	Duration: seven minutes, twice daily
	To elongate the soft tissue	Isolated stretching of the gastrocnemius muscles and the soleus-Achilles is done with the knee extended (gastrocnemius) or flexed (soleus-Achilles)	Five repetitions × one set (30-second hold)
	To improve strength for ankle and foot muscles	Elastic bands and closed chain exercises: seated calf pumps, bipedal calf pumps, and single-leg calf pump	Ten repetitions × one set (two times per day)
	To improve the ROM ankle joint	Pain-free PROM, AAROM, and AROM exercises for the ankle joint	Ten repetitions × one set (10-second hold)
		Wall-supported squats and lunges	
	To improve balance and proprioception	Ankle PNF (contract-relax method)	Ten repetitions × one set (two times per day)
		Spot marching	
		Weight shifts on a Swiss ball	
		Wobble board balancing exercises	
	To improve gait patterns	Walking with the support of a walker, progressing to independent ambulation	Two rounds (two times per day)

Follow-up and outcome measures

The patient underwent an organized physical therapy protocol for four weeks, and a follow-up was carried out. The findings of the outcome measure are shown in Tables 2-4.

**Table 2:** Pre- and post-physiotherapeutic rehabilitation muscular strength 2, full ROM with gravity eliminated; 4, full ROM against gravity, moderate resistance MMT, manual muscle testing; ROM, range of motion

Muscle group	Pre-treatment MMT grade	Post-treatment MMT grade
Dorsiflexors	2	4
Plantar flexors	2	4
Invertors	2	4
Evertors	2	4

**Table 3:** Pre- and post-physiotherapeutic rehabilitation ROM ROM, range of motion

Movement	Pre-treatment		Post-treatment	
	Active	Passive	Active	Passive
Dorsiflexion	0-5⁰	0-15⁰	0-18⁰	0-20⁰
Plantarflexion	0-10⁰	0-15⁰	0-40⁰	0-45⁰
Inversion	0-5⁰	0-10⁰	0-30⁰	0-32⁰
Eversion	0-5⁰	0-8⁰	0-12⁰	0-15⁰

**Table 4:** Pre- and post-physiotherapeutic rehabilitation outcome measures F&A, foot and ankle; FIM, functional independence measure; NPRS, Numerical Pain Rating Scale

Outcome measures	Pre-intervention	Post-intervention
F&A Disability Index	20/104	98/104
Cumberland Ankle Instability Tool	Jun-30	27/30
Dynamic Gait Index	May-24	22/24
NPRS	On activity: 4/10	On activity: 2/10
	On rest: 3/10	On rest: 1/10

## Discussion

Despite being the most commonly ruptured tendon in the human body, the AT has a relatively unknown etiology. The most effective approach to treating total rupture is still up for debate despite an ample number of published studies. The treatment can be broadly classified into non-operative and operative. Post-operative physiotherapy management is necessary to gain early functional recovery and prevent secondary complications. In this study, we have discussed a case of a 45-year-old female diagnosed with AT rupture and managed surgically with soft tissue repair. The AT reconstruction led to post-operative pain and loss of ankle proprioception, ROM, and strength, for which she had undergone physiotherapy management, which proved to be effective in early functional recovery.

Ankle proprioception is a vital component of balance because it provides information that is required to modify ankle position during multifaceted motor tasks. Improving ankle proprioception was our main goal in order to promote an early functional recovery. Therefore, we included exercises including weight shifts on a Swiss ball, wobble board balancing, and ankle proprioceptive neuromuscular facilitation techniques using the contract-relax approach. Early enhancements in joint proprioception were revealed by these interventions, which aided in earlier mobilization. In a trial including participants with chronic ankle injuries, Alahmari et al. similarly demonstrated progressive strengthening and proprioceptive training methods, resulting in significant improvements in proprioception, stability, self-reported functional status, and balance [15]. Ankle proprioceptive exercises enhance eversion, plantarflexion, dorsiflexion, and inversion joint position sense in athletes with functional ankle instability, according to research by Singh et al. [16]. We gave cryotherapy to reduce post-operative pain. According to Khadijah et al., a specific type of transcendence that has been attained in cold compresses to make individuals feel more comfortable is the evolution of pain perception to a more dominating experience of cold. Consequently, it can be considered that cryotherapy is superior to warm compresses in terms of lowering pain perception and enhancing comfort, based on current theories and evidence [17].

A range of varied exercise-based interventions were used in early functional rehabilitation protocols. The most often used intervention was ankle ROM, which was followed by strengthening exercises. The purpose of ROM is probably to promote tendon gliding and to avoid deep adhesion, both of which aid in the early acquisition of mobility [18].

## Conclusions

A key component of AT rupture treatment is choosing the best post-operative rehabilitation regimen that emphasizes functional physiotherapy components. Improved functional outcomes following surgery are the result of carefully planned rehabilitation practices. Combining surgical intervention with appropriate rehabilitation measures facilitates the restoration of ankle mobility in individuals undergoing heel cord reconstruction. In this case report, we saw that the patient had improved ankle proprioception, muscular strength, and ROM, which led to an early functional recovery. Proprioceptive training shortened the period of recovery and enabled the patient to regain full control over the joint.
